# Risk of major bleeding by ethnicity and socioeconomic deprivation among 488,107 people in primary care: a cohort study

**DOI:** 10.1186/s12872-021-01993-9

**Published:** 2021-04-23

**Authors:** Wai Chung Tse, Corina Grey, Matire Harwood, Rod Jackson, Andrew Kerr, Suneela Mehta, Katrina Poppe, Romana Pylypchuk, Sue Wells, Vanessa Selak

**Affiliations:** 1 School of Medicine, Monash University, Clayton, Australia; 2 Section of Epidemiology and Biostatistics, University of Auckland, Private Bag 92019, Auckland, 1142 New Zealand; 3 General Practice and Primary Health Care, University of Auckland, Auckland, New Zealand; 4 Middlemore Hospital, Auckland, New Zealand

**Keywords:** Cardiovascular disease, Bleeding risk, Ethnicity, Socioeconomic status

## Abstract

**Background:**

Antithrombotic medications (antiplatelets and anticoagulants) reduce the risk of cardiovascular disease (CVD), but with the disadvantage of increasing bleeding risk. Ethnicity and socioeconomic deprivation are independent predictors of major bleeds among patients without CVD, but it is unclear whether they are also predictors of major bleeds among patients with CVD or atrial fibrillation (AF) after adjustment for clinical variables.

**Methods:**

Prospective cohort study of 488,107 people in New Zealand Primary Care (including 64,420 Māori, the indigenous people of New Zealand) aged 30–79 years who had their CVD risk assessed between 2007 and 2016. Participants were divided into three mutually exclusive subgroups: (1) AF with or without CVD (n = 15,212), (2) CVD and no AF (n = 43,790), (3) no CVD or AF (n = 429,105). Adjusted hazards ratios (adjHRs) were estimated from Cox proportional hazards models predicting major bleeding risk for each of the three subgroups to determine whether ethnicity and socioeconomic deprivation are independent predictors of major bleeds in different cardiovascular risk groups.

**Results:**

In all three subgroups (AF, CVD, no CVD/AF), Māori (adjHR 1.63 [1.39–1.91], 1.24 [1.09–1.42], 1.57 [95% CI 1.45–1.70], respectively), Pacific people (adjHR 1.90 [1.58–2.28], 1.30 [1.12–1.51], 1.62 [95% CI 1.49–1.75], respectively) and Chinese people (adjHR 1.53 [1.08–2.16], 1.15 [0.90–1.47], 1.13 [95% CI 1.01–1.26], respectively) were at increased risk of a major bleed compared to Europeans, although for Chinese people the effect did not reach statistical significance in the CVD subgroup. Compared to Europeans, Māori and Pacific peoples were generally at increased risk of all bleed types (gastrointestinal, intracranial and other bleeds). An increased risk of intracranial bleeds was observed among Chinese and Other Asian people and, in the CVD and no CVD/AF subgroups, among Indian people. Increasing socioeconomic deprivation was also associated with increased risk of a major bleed in all three subgroups (adjHR 1.07 [1.02–1.12], 1.07 [1.03–1.10], 1.10 [95% CI 1.08–1.12], respectively, for each increase in socioeconomic deprivation quintile).

**Conclusion:**

Ethnicity and socioeconomic status should be considered in bleeding risk assessments to guide the use of antithrombotic medication for the management of AF and CVD.

**Supplementary Information:**

The online version contains supplementary material available at 10.1186/s12872-021-01993-9.

## Background

Antithrombotic medications (antiplatelets and anticoagulants) reduce the risk of cardiovascular disease (CVD), but with the disadvantage of increasing bleeding risk. Bleeding risk equations have been developed so that clinicians can balance the potential cardiovascular benefits of antithrombotic medication against their bleeding harms. We recently developed such an equation for people without CVD or atrial fibrillation (AF) to guide decision-making on the use of aspirin for the primary prevention of CVD [1, 2]. We found that ethnicity and socioeconomic status, in addition to clinical variables, were important independent predictors of major bleeds among women and men.

Neither ethnicity nor socioeconomic status are specified as factors associated with increased bleeding risk in US guidelines for the secondary prevention of CVD [3] or management of AF [4]. Bleeding risk is of particular importance among people with AF given the widespread use of anticoagulants to reduce the risk of thromboembolic disease, especially stroke, in this group. The purpose of this study was to determine whether ethnicity and/or socioeconomic status are also independent risk factors for a major bleed among people with a history of CVD or AF.

## Methods

### Setting

This study was a prospective open cohort study. People were entered into the cohort the first time their primary care physician or nurse entered their CVD risk assessment data into PREDICT, a web-based decision support program integrated with electronic primary care practice management systems in New Zealand [5]. More than one third of primary care practices in New Zealand use the PREDICT software. In these practices, data up to 2015 indicate that approximately 90% of persons eligible for CVD risk assessment (according to national guidelines [6]) had their risk assessed using this software [5]. Participants were recruited between 1 January 2007 and 31 December 2016. The study end date was 31 December 2017, which provided at least 12 months of follow-up across all data sources. Participants were censored on the earliest of the following dates: death, 5 years follow up or the study end date.

Since 2003, New Zealand CVD risk management guidelines have recommended a regular CVD risk assessment for men aged 45 years or older, women aged 55 years or older (and 10 years earlier for subpopulations at increased risk: those of Māori, Pacific, or Indian ethnicity) [7]. Whether a person visiting the primary care clinic has his or her CVD risk assessed, and therefore whether he or she enters the cohort, is at the discretion of the primary care clinician.

### Data sources and Linkage

During CVD risk assessment, an electronic risk profile is stored both in the practice management system and anonymously on a central database. With the permission of clinicians, this profile is linked to an encrypted National Health Index number, which is used to anonymously link the profile to national (mortality, hospitalisation and medication dispensing) and regional (laboratory result) databases.

### Inclusion and exclusion criteria

All people who had a first CVD risk assessment in primary care using the PREDICT program between 1 January 2007 and 31 December 2016 were considered for inclusion in this study. Exclusion criteria were any of the following at the time of risk assessment: age younger than 30 years or age 80 years or older, history of intracranial haemorrhage (ICH, as antithrombotic therapy is generally contraindicated in this group), or with ethnicity of MELAA (Middle Eastern/Latin American/African) or Other (due to the small numbers in these ethnic groups in New Zealand). Participants were divided into three mutually exclusive subgroups: AF (whether or not they had CVD), CVD (without AF), and without either AF or CVD. Definitions of AF, CVD, intracranial bleeding and ethnicity are provided in Additional file 1: Table S1.

### Outcome

The primary outcome in this study was time to a first major bleeding event (hospitalization or death associated with bleeding) during follow up (any, or for sensitivity analyses by type: gastrointestinal, intracranial, other). Hospitalizations with bleeding were defined as those in which an International Statistical Classification of Diseases and Related Health Problems (ICD) code for a bleeding event (Additional file 1: Table S2) was assigned as a diagnosis for the admission, either on its own if it was the principal diagnosis (i.e., the main reason for the admission) or, if the bleed was not the principal diagnosis, when there was also a transfusion of whole blood (code 1370601 in the ICD, Tenth Revision, Australian Modification [ICD-10-AM]; code 9903 in the Australian version of the ICD, 9th Revision, Clinical Modification [ICD-9-CM-A]) or a blood transfusion of packed cells [ICD-10-AM code 1370602; ICD-9-CM-A code 9904]). Potential ICD codes for a major bleed were identified by a review of ICD code sets used by other studies to identify bleeding events [8–11] and a review (by VS) of all ICD-9-CM-A and ICD-10-AM codes for any additional potentially relevant codes. The final set of ICD-9-CM-A and ICD-10-AM codes for a major bleed (Additional file 1: Table S2) was compiled following a review of all potential ICD codes by VS and AK. Deaths with bleeding were defined as those in which an ICD code for a bleeding event (Additional file 1: Table S2) was the underlying cause of death. Major bleeding associated with trauma or procedures was excluded.

### Variables

To classify ethnicity, we used self-reported ethnicity categorised using the prioritised output method of national ethnicity data protocols [12] with the Indian population (who comprise 90% of the South Asian population in New Zealand [13]) separated out from the Asian category as they are known to have elevated CVD risk. Non-Indian South Asians cannot currently be distinguished from other Asians in New Zealand administrative health data. This classified the population into Māori (the indigenous people of New Zealand), Pacific, Indian, Chinese, Other Asian, European, MELAA and Other ethnicity.

Socioeconomic deprivation was measured using an area-based measure, the New Zealand Index of Deprivation (NZDep) [14]. The NZDep was constructed from nine census derived variables representing eight dimensions of deprivation. Deprivation was classified in quintiles from 1 (least deprived) to 5 (most deprived).

The following predictors were also included in the analysis: age, sex, systolic blood pressure (SBP), total cholesterol to high density lipoprotein cholesterol ratio (TC:HDL), medical history (smoking, diabetes, CVD, heart failure, cancer, bleeding, peptic ulcer disease, thrombocytopaenia, anaemia, chronic kidney disease, liver disease, pancreatitis or alcohol-related conditions) and medication use (antiplatelet, anticoagulant, antihypertensive, lipid lowering, non-steroidal anti-inflammatory, corticosteroid, selective serotonin reuptake inhibitor). Medications included within each of these drug classes (listed in Additional file 1: Table S3) were all of those with approval for use in New Zealand at the time of the study. For antiplatelets these were aspirin, clopidogrel, dipyridamole, prasugrel, ticagrelor and ticlopidine. For anticoagulants these were dabigatran, phenindione, rivaroxaban and warfarin.

Definitions of all predictors are provided in Additional file 1: Table S1.

### Statistical analyses

Continuous variables were summarized as means with standard deviations and medians with interquartile ranges (IQR) and categorical data as frequencies with percentages. Adjusted hazards ratios (adjHRs) for time to a first major bleed during follow up were obtained using Cox proportional hazards models. All variables noted above were included. Separate models were developed in each of the three subgroups for all bleeds. In sensitivity analyses, separate models were developed in each of the three subgroups for three bleed types: gastrointestinal, intracranial, other. Time in the study was the time scale and was calculated from index assessment to the earliest of the following dates: first major bleed during follow up, death, 5 years follow up or the study end date (31 December 2017). Reference groups for categorical variables are shown in bold and underlined in Additional file 1: Table S1. The proportionality assumption in each model was assessed by using the global Schoenfeld test [15] and plotting log[− log(survival)] versus log(time). The linearity of the association between continuous variables (age, SBP and TC:HDL) and the outcome was assessed by visual inspection of LOWESS smoothed plots of martingale residuals [16]. Data analysis was performed using R software, version 3.5.1 (https://cran.r-project.org/), which included the “survival” package.

## Results

**Fig. 1:**
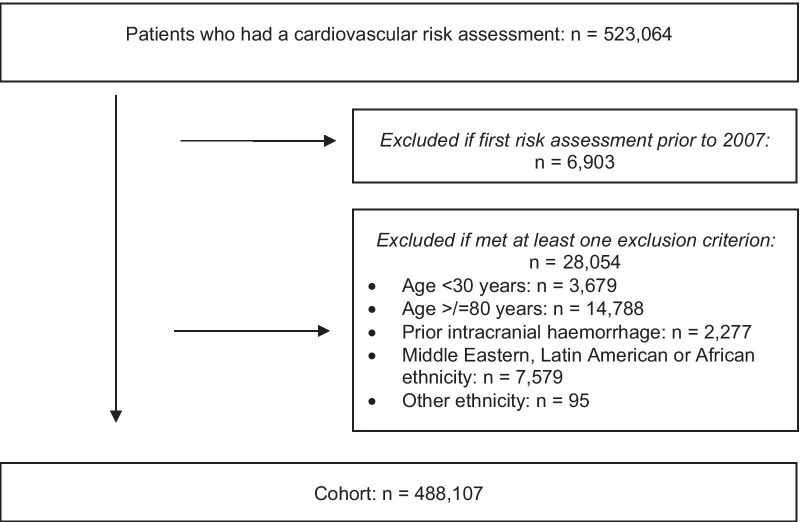
Cohort enrolment, exclusions

A total of 523,064 people who had a PREDICT CVD risk assessment were considered for the analysis (Fig. 1). Of these, 6,903 people were excluded because their first risk assessment was prior to 2007. Among the remaining 516,161 people, 28,054 were excluded because they met at least one of the exclusion criteria.

Among the remaining cohort of 488,107 people, 56.4% were men and the median age was 55 years (IQR 47–63). Although the majority were European (272,499, 55.8%), there were large numbers in the other ethnic groups: 64,420 (13.2%) Māori, 60,905 (12.5%) Pacific, 41,505 (8.5%) Indian, 28,948 (5.9%) Chinese and 19,830 (4.1%) Other Asian. There was a higher proportion of people living in areas of the highest (22.3%) and lowest (21.6%) quintiles of socioeconomic deprivation, than those living in intermediate quintiles (second quintile 19.4%, third quintile 18.0%, fourth quintile 18.7%).

We identified a total of 15,212 (3.1%), 43,790 (9.0%), and 429,105 (87.9%) people with AF (± CVD), CVD (no AF), and no CVD or AF, respectively (Table 1). There was a greater proportion of men, Europeans and Māori in those with AF or CVD compared with those without either. Those with AF or CVD were also older and more likely to be in the most deprived quintile of socioeconomic deprivation than those without either. The median follow-up (5.0 years, IQR 3.8–5.0) was similar across subgroups. During follow up there were a total of 9,873 bleeds (AF 1,218, CVD 2,105, no CVD/AF 6550), of which 6736 were gastrointestinal, 1384 were intracranial and 2024 were other bleeds. Cumulative incidence of bleeds (total and by type) for each of the subgroups is plotted in eFigure 1.

**Table 1:** Baseline characteristics, follow up duration and bleeds during follow up, by subgroup

Variables	Atrial fibrillation	Cardiovascular disease	No atrial fibrillation or cardiovascular disease
n (% of total)	15,212 (3.1%)	43,790 (9.0%)	429,105 (87.9%)
Male	9857 (64.8%)	25,704 (58.7%)	239,552 (55.8%)
Age, years	64 ± 10 (66, 58 to 72)	62 ± 910 (64, 56 to 70)	54 ± 10 (54, 46 to 61)
Ethnicity (self-identified and prioritised)			
Māori	2885 (19%)	6452 (14.7%)	55,083 (12.8%)
Pacific	1578 (10.4%)	4671 (10.7%)	54,656 (12.7%)
Indian	340 (2.2%)	3226 (7.4%)	37,939 (8.8%)
Chinese	428 (2.8%)	1526 (3.5%)	26,994 (6.3%)
Other Asian	158 (1%)	909 (2.1%)	18,763 (4.4%)
European	9823 (64.6%)	27,006 (61.7%)	235,670 (54.9%)
Socioeconomic deprivation*			
Quintile 1 (least deprived)	2953 (19.4%)	7815 (17.8%)	94,741 (22.1%)
Quintile 2	2735 (18%)	7585 (17.3%)	84,564 (19.7%)
Quintile 3	2697 (17.7%)	7832 (17.9%)	77,327 (18%)
Quintile 4	2956 (19.4%)	9046 (20.7%)	79,204 (18.5%)
Quintile 5 (most deprived)	3871 (25.4%)	11,512 (26.3%)	93,269 (21.7%)
Measurements			
Systolic blood pressure, mm Hg	130 ± 16 (130, 120 to 140)	132 ± 16 (130, 122 to 141)	129 ± 16 (128, 120 to 138)
TC:HDL, mmol/L	3.8 ± 1.2 (3.6, 3.0 to 4.5)	3.8 ± 1.2 (3.6, 3.0 to 4.5)	4.1 ± 1.2 (3.9, 3.2 to 4.8)
Medical history			
Smoker (current or former)	6011 (39.5%)	18,725 (42.8%)	134,420 (31.3%)
Diabetes	3942 (25.9%)	13,594 (31%)	51,497 (12%)
Coronary heart disease	5598 (36.8%)	28,219 (64.4%)	0 (0%)
Percutaneous coronary intervention or coronary artery bypass graft	2614 (17.2%)	12,644 (28.9%)	0 (0%)
Cerebrovascular disease	2070 (13.6%)	9894 (22.6%)	0 (0%)
Peripheral vascular disease	1588 (10.4%)	6387 (14.6%)	0 (0%)
Heart failure	4889 (32.1%)	9777 (22.3%)	0 (0%)
Atrial fibrillation	15,212 (100%)	0 (0%)	0 (0%)
Cancer	1899 (12.5%)	4517 (10.3%)	22,330 (5.2%)
Gastrointestinal bleed	1373 (9%)	2726 (6.2%)	8173 (1.9%)
Other^†^ bleed	895 (5.9%)	1278 (2.9%)	2921 (0.7%)
Peptic ulcer disease	4749 (31.2%)	14,499 (33.1%)	56,353 (13.1%)
Thrombocytopenia	854 (5.6%)	1610 (3.7%)	6694 (1.6%)
Anaemia	1794 (11.8%)	4566 (10.4%)	14,352 (3.3%)
Chronic kidney disease	666 (4.4%)	1885 (4.3%)	2148 (0.5%)
Chronic liver disease	94 (0.6%)	286 (0.7%)	752 (0.2%)
Chronic pancreatitis	33 (0.2%)	82 (0.2%)	270 (0.1%)
Chronic alcohol-related disease	467 (3.1%)	901 (2.1%)	2931 (0.7%)
Medication (preceding 6 months)			
Antiplatelet	6928 (45.5%)	29,329 (67%)	41,743 (9.7%)
Dual antiplatelet	442 (2.9%)	3722 (8.5%)	190 (0.0%)
Anticoagulant	6265 (41.2%)	1207 (2.8%)	1244 (0.3%)
Blood pressure-lowering	12,089 (79.5%)	33,027 (75.4%)	101,896 (23.7%)
Lipid-lowering	7861 (51.7%)	30,072 (68.7%)	71,831 (16.7%)
Non-steroidal anti-inflammatory	2011 (13.2%)	7710 (17.6%)	77,586 (18.1%)
Steroid	1813 (11.9%)	4585 (10.5%)	23,505 (5.5%)
Selective serotonin re-uptake inhibitor	1017 (6.7%)	3586 (8.2%)	22,192 (5.2%)
Follow-up duration			
Total, person-years	62,657	189,336	1,822,153
Median (interquartile range), years	5.0 (3.6 to 5.0)	5.0 (4.1 to 5.0)	4.9 (3.7 to 5.0)
Major bleeds during follow up			
Any	1218 (8.0%)	2105 (4.8%)	6550 (1.5%)
Gastrointestinal	734 (4.8%)	1476 (3.4%)	4526 (1.1%)
Intracranial	172 (1.1%)	273 (0.6%)	939 (0.2%)
Other^†^	384 (2.5%)	428 (1.0%)	1212 (0.3%)

*HDL* high density lipoprotein, *TC* total cholesterol

Categorical data are n (%) of subgroup, unless indicated otherwise

Continuous data are mean ± standard deviation (median, interquartile range), unless indicated otherwise

Data complete or near complete (> 99% of values available)

*Socioeconomic deprivation measured by the New Zealand Deprivation Index (2006), an area-based measure constructed from 9 census derived variables representing 8 dimensions of deprivation

^†^Other bleeds were respiratory bleeds (including epistaxis and haemoptysis), ocular bleeds (vitreous and retinal), bleeds into a joint and bleeds into the pericardium or peritoneum

The proportionality assumption and linearity of the association between continuous variables (age, SBP, TC:HDL) and the outcomes were assessed for the three main models (outcome = all bleeds) and the nine models in the sensitivity analysis (outcome = gastrointestinal bleeds, intracranial bleeds, other bleeds). The proportionality assumption largely held for all variables across each of the models. While the association between age and TC:HDL and the outcomes was largely linear, there was some non-linearity in the association between SBP and the outcomes in some of the models at 140 mmHg, and therefore SBP was dichotomised for all models (< 140, ≥ 140 mmHg).

The adjHRs for ethnicity and socioeconomic deprivation in all of the models (main and sensitivity) are provided in Tables 2 and 3, respectively, and the adjHRs for all variables in each of the subgroups are provided in Additional file 1: Table S4 (main) and Additional file 1: Tables S5–S7 (sensitivity). The adjHRs for ethnicity in all of the models are also presented as forest plots in eFigure 2. In all three subgroups (AF, CVD, no CVD or AF), Māori (adjHR 1.63 [1.39–1.91], 1.24 [1.09–1.42], 1.57 [95% CI 1.45–1.70], respectively), Pacific people (adjHR 1.90 [1.58–2.28], 1.30 [1.12–1.51], 1.62 [95% CI 1.49–1.75], respectively) and Chinese people (adjHR 1.53 [1.08–2.16], 1.15 [0.90–1.47], 1.13 [95% CI 1.01–1.26], respectively) were at increased risk of a major bleed compared to Europeans, although for Chinese people the effect did not reach statistical significance in the CVD subgroup (Table 2). For Māori and Pacific people there was a fairly consistent increase in bleeding risk compared with Europeans across the three subgroups for each of the three bleed types. In contrast, for Chinese people the increase in bleeding risk in the three subgroups was mainly due to an increased risk of intracranial bleeds compared with Europeans. There was also an increased risk of intracranial bleeds among Other Asian people and, in the CVD and no CVD/AF subgroups, among Indian people.

**Table 2:** Adjusted hazard ratios for ethnicity, by subgroup and bleed type

Ethnicity (self-identified and prioritised; comparator European)	Atrial fibrillation	Cardiovascular disease	No atrial fibrillation or cardiovascular disease
*Any bleed*			
Māori	1.63 (1.39–1.91)	1.24 (1.09–1.42)	1.57 (1.45–1.70)
Pacific	1.90 (1.58–2.28)	1.30 (1.12–1.51)	1.62 (1.49–1.75)
Indian	0.75 (0.48–1.19)	0.98 (0.82–1.19)	0.95 (0.85–1.06)
Chinese	1.53 (1.08–2.16)	1.15 (0.90–1.47)	1.13 (1.01–1.26)
Other Asian	1.22 (0.65–2.29)	1.05 (0.75–1.46)	1.34 (1.17–1.52)
*Gastrointestinal bleed*			
Māori	1.48 (1.21–1.82)	1.17 (1.00–1.38)	1.47 (1.34–1.62)
Pacific	1.64 (1.29–2.08)	1.08 (0.90–1.30)	1.51 (1.37–1.66)
Indian	0.59 (0.31–1.11)	0.93 (0.75–1.16)	0.93 (0.81–1.06)
Chinese	1.43 (0.92–2.22)	1.06 (0.78–1.43)	1.03 (0.90–1.17)
Other Asian	1.00 (0.41–2.43)	0.93 (0.62–1.41)	1.24 (1.06–1.45)
*Intracranial bleed*			
Māori	1.49 (0.97–2.30)	0.96 (0.63–1.47)	1.44 (1.16–1.78)
Pacific	1.97 (1.23–3.16)	2.09 (1.43–3.05)	1.73 (1.40–2.14)
Indian	1.00 (0.36–2.79)	1.67 (1.05–2.64)	1.38 (1.05–1.82)
Chinese	3.51 (1.79–6.87)	1.77 (0.99–3.16)	1.45 (1.12–1.89)
Other Asian	2.60 (0.81–8.32)	2.08 (1.01–4.29)	1.67 (1.21–2.30)
*Other bleed*			
Māori	1.98 (1.50–2.61)	1.52 (1.15–2.03)	2.28 (1.92–2.70)
Pacific	2.35 (1.71–3.24)	1.65 (1.22–2.24)	2.05 (1.71–2.46)
Indian	0.94 (0.44–2.04)	0.86 (0.55–1.34)	0.73 (0.54–0.98)
Chinese	0.75 (0.31–1.82)	1.17 (0.67–2.07)	1.33 (1.03–1.71)
Other Asian	0.78 (0.19–3.17)	0.62 (0.23–1.68)	1.47 (1.09–1.99)

Hazard ratios are adjusted for all of the following variables: age, sex, ethnicity, socioeconomic deprivation, systolic blood pressure (> / = or < 140 mm Hg), ratio of total cholesterol to high density lipoprotein cholesterol (mmol/L), medical history (smoking, diabetes, cardiovascular disease, heart failure, cancer, bleeding, peptic ulcer disease, thrombocytopaenia, anaemia, chronic kidney disease, liver disease, pancreatitis or alcohol-related condition) and medication use in the preceding 6 months (antiplatelet, anticoagulant, BP-lowering, lipid-lowering, non-steroidal anti-inflammatory, steroid, selective serotonin re-uptake inhibitor)

The total number of people included (and excluded due to a missing value) in the models for atrial fibrillation, cardiovascular disease and no atrial fibrillation or cardiovascular disease were: 15,097 (115), 43,437 (353) and 425,668 (3437), respectively

For the atrial fibrillation subgroup, the number of bleeds in the models were: any (1202), gastrointestinal (723), intracranial (172), other (379). For the cardiovascular disease subgroup, the number of bleeds in the models were: any (2092), gastrointestinal (1470), intracranial (270), other (423). For the no atrial fibrillation or cardiovascular disease subgroup, the number of bleeds in the models were: any (6478), gastrointestinal (4482), intracranial (926), other (1196)

**Table 3:** Adjusted hazard ratios for socioeconomic deprivation, by subgroup and bleed type

Socioeconomic deprivation	Atrial fibrillation	Cardiovascular disease	No atrial fibrillation or cardiovascular disease
Area-based, per quintile of increasing deprivation, compared with those living in the quintile of least deprivation	*Any bleed*		
	1.07 (1.02–1.12)	1.07 (1.03–1.10)	1.10 (1.08–1.12)
	*Gastrointestinal bleed*		
	1.07 (1.01–1.13)	1.09 (1.04–1.13)	1.11 (1.08–1.13)
	*Intracranial bleed*		
	1.23 (1.09–1.40)	1.00 (0.91–1.10)	1.07 (1.02–1.12)
	*Other bleed*		
	1.01 (0.93–1.09)	1.04 (0.96–1.12)	1.08 (1.03–1.13)

Hazard ratios are adjusted for all of the following variables: age, sex, ethnicity, socioeconomic deprivation, systolic blood pressure (> / = or < 140 mm Hg), ratio of total cholesterol to high density lipoprotein cholesterol (mmol/L), medical history (smoking, diabetes, cardiovascular disease, heart failure, cancer, bleeding, peptic ulcer disease, thrombocytopaenia, anaemia, chronic kidney disease, liver disease, pancreatitis or alcohol-related condition) and medication use in the preceding 6 months (antiplatelet, anticoagulant, BP-lowering, lipid-lowering, non-steroidal anti-inflammatory, steroid, selective serotonin re-uptake inhibitor)

The total number of people included (and excluded due to a missing value) in the models for atrial fibrillation, cardiovascular disease and no atrial fibrillation or cardiovascular disease were: 15,097 (115), 43,437 (353) and 425,668 (3437), respectively

For the atrial fibrillation subgroup, the number of bleeds in the models were: any (1202), gastrointestinal (723), intracranial (172), other (379). For the cardiovascular disease subgroup, the number of bleeds in the models were: any (2092), gastrointestinal (1470), intracranial (270), other (423). For the no atrial fibrillation or cardiovascular disease subgroup, the number of bleeds in the models were: any (6478), gastrointestinal (4482), intracranial (926), other (1196)

Increasing socioeconomic deprivation was associated with increased risk of a major bleed in all three subgroups (adjHR 1.07 [1.02–1.12], 1.07 [1.03–1.10], 1.10 [95% CI 1.08–1.12], respectively, for each increase in socioeconomic deprivation quintile) (Table 3). There was a fairly consistent increase in bleeding risk with increasing socioeconomic deprivation across the three subgroups for each of the three bleed types.

## Discussion

We found that, after adjusting for multiple clinical variables, ethnicity and socioeconomic deprivation were independent predictors of major bleeds in those with AF, CVD and without either condition. Māori and Pacific people were at increased risk compared with Europeans across most bleed types / subgroups, whereas Indian, Chinese and Other Asians were at increased risk of intracranial bleeds compared with Europeans across most subgroups. Increasing socioeconomic deprivation was also associated with a higher risk of a major bleed across most bleed types / subgroups.

Strengths of this study are its considerable size overall and the substantial size of the included ethnic and socioeconomic groups. The size of the cohort overall and its heterogeneity enabled us to adequately address the research question by simultaneously adjusting for multiple demographic and clinical variables in distinct clinical cohorts.

There are a number of limitations to this study. The study is reliant on coded diagnoses, rather than clinical diagnoses, to identify major bleeds. This definition, though informed by clinicians (including a cardiologist, AK) and previous literature, and used in our previously published work [1, 2, 17], has not been validated against source documentation for sensitivity in identifying bleeding events. While some major bleeds may have been missed or misclassified in this study due to the reliance on coded data, this limitation is unlikely to have a substantive effect on our findings because it is unlikely that coding varies by ethnicity or socioeconomic deprivation. Another limitation of the study is that it was restricted to patients who had their CVD risk assessed. However, in included practices, data up to 2015 indicate that a CVD risk assessment had been conducted in approximately 90% of eligible persons [5]. Finally, while participants were censored at death, competing risk of non-bleeding death was not taken into account when risk of bleeding was estimated.

While we have noted the large sample size as a strength, a potential disadvantage of large sample size is the increased likelihood of identifying statistically significant differences between groups which may not have clinical relevance. We consider the magnitude of the independent effect of ethnicity and socioeconomic status on bleeding risk (which has taken into account multiple other predictors) to be clinically, as well as statistically significant. For example, after taking all other predictors into account, and depending on the specific clinical subgroup, Māori were 24–63% and Pacific people 30–90% more likely to have a major bleed than Europeans, and a person living in an area with the highest quintile of socioeconomic deprivation was 35–50% more likely to have a major bleed than a person living in an area with the lowest quintile of socioeconomic deprivation.

This study was reliant on secondary use of patient data, i.e. data that had already been collected. Further, in this study, patient data had already been collected through the process of health service delivery, and without imposing any additional burden on patients, clinicians or health services. It would have been impossible within our resource constraints (and those of any other research or health provider group in this and probably other countries) to obtain informed consent from included participants given the size of the study, which as we have noted above, was needed because our analyses required simultaneous adjustment for multiple demographic and clinical variables in distinct clinical cohorts. A waiver was granted by the national ethics committee to enable this and other similar research to proceed with their permission without obtaining informed consent from participants, because the study involved secondary use of routinely collected patient data, and patient data were anonymized prior to being received by the research team.

Our findings are largely consistent with those of other New Zealand [18–20] and international [21–40] studies that have also found higher rates of major bleeds (total, gastrointestinal or intracranial) in non-Whites compared with Whites and with increasing socioeconomic deprivation. In these studies, populations were not defined according to their history of CVD and AF, and there was adjustment for a limited number of variables, if any. Studies of people with AF have shown this group to have increased risk of major bleeds with more deprived socioeconomic status [41], and increased risk of intracranial haemorrhage if they were Asian, Hispanic or Black (compared with White people) [42].

We identified two studies with findings inconsistent with our study [43, 44]. A systematic review of population-based studies of intracranial haemorrhage that pooled incidences in a random-effects binomial meta-analysis found that the incidence of intracranial haemorrhage was greater for East and Southeast Asian people than for White people, whereas there was no statistically significant differences in incidence for Black, Hispanic, Indian or Māori compared with White people [43]. There are a number of significant limitations to this study, including inconsistency in case definition and ascertainment across included studies and the inability to adjust for individual participant characteristics such as age because of a reliance on aggregated data [43].

The QBleed sex-specific risk equations for upper gastrointestinal and intracranial bleeds were developed among people in primary care irrespective of their history of CVD or AF, using multiple variables from routinely collected data from general practices in the United Kingdom [44]. Increasing socioeconomic deprivation was associated with an increase in bleeds, both upper gastrointestinal and intracranial. In QBleed there was no statistically significant difference in the risk of upper gastrointestinal bleeds between those in any of the ethnic groups (Indian, Pakistani, Bangladeshi, Other Asian, Caribbean, Black African, Chinese, Other [including mixed]) compared with the reference group (Whites or people in whom ethnicity was not recorded), with the exception of Black African men who were at lower risk of an upper gastrointestinal bleed than the reference group [44]. The following ethnic groups were at increased risk of an intracranial bleed: Bangladeshi women, Other Asian men, Caribbean men and Black African men. Chinese people were at increased risk of an intracranial bleed but the difference was not statistically significant. There are a number of reasons that might explain why differences by ethnicity were more consistently observed in our study than in QBleed. Ethnicity was missing from 26% of the QBleed participants, the reference ethnic group in QBleed combined Whites and those in whom ethnicity was missing, and the proportions in the non-reference groups was low (Indian 2.1%, Pakistani 1.0%, Bangladeshi 0.8%, Other Asian 1.4%, Caribbean 1.0%, Black African 1.8%, Chinese 1.1% and other or multiple 2.3%).

We consider that a plausible explanation for the finding of higher rates of major bleeds in non-Whites compared with Whites and with increasing socioeconomic deprivation to be that ethnicity and socioeconomic deprivation are surrogate markers for longitudinal, lifetime exposure to risk factors that are not able to be captured by other predictors either because these are cross sectional or not measured at all. Ethnicity itself has been directly associated with adverse health outcomes through racism [45, 46].

These findings indicate that clinical decision-making regarding the use of antithrombotic medication for the management of AF and CVD, as well as the primary prevention of CVD, should take ethnicity and socioeconomic deprivation into account. In order to be able to do this, clinicians need multivariable bleeding risk assessment tools. Clinicians need such tools to enable them to take into account ethnicity and socioeconomic deprivation at the same time as other independent predictors of bleeding risk. Ideally such bleeding risk assessment tools would be validated in the local population.

US guidelines for the secondary prevention of CVD recommend antiplatelet therapy in specific groups, largely irrespective of bleeding risk [3]. More recent US and European post-acute coronary syndrome guidelines [47, 48], have moved towards the use of multivariable equations to estimate the risk of bleeds, but none of these equations include ethnicity or socioeconomic status [49–51]. Similarly, while US guidelines for the management of AF [4] recommend a multivariable equation to estimate the risk of bleeds, that equation [52] also does not include ethnicity or socioeconomic status.

Given the significant independent effects of socioeconomic status and ethnicity on major bleeds, it would be appropriate for future guidelines to consider both factors in bleeding risk assessments, ideally using multivariable equations, to guide the use of antithrombotic medication for the management of AF and CVD.

## Conclusion

We found that ethnicity and socioeconomic deprivation were independent predictors of major bleeds in those with AF, CVD and without either condition. Clinical decisions regarding the use of antithrombotic medication for the management of AF and CVD, as well as the prevention of CVD, should take ethnicity and socioeconomic deprivation into account. Clinicians need locally validated multivariable bleeding risk assessment tools to enable them to take into account ethnicity and socioeconomic deprivation, along with other independent predictors, when considering and discussing the balance of benefits and harms of antithrombic medication with their patients.

## Supplementary Information

**Additional file1: Table S1**. Definitions of variables. **Table S2**. ICD codes used to identify medical history or outcomes from hospital or death records. **Table S3**. Medications included in drug classes. **Table S4**. Adjusted hazard ratios for any bleed, by subgroup. **Table S5**. Adjusted hazard ratios for gastrointestinal bleed. **Table S6**. Adjusted hazard ratios for intracranial bleed. **Table S7**. Adjusted hazard ratios for other bleed. **eFigure 1**. Cumulative incidence of a bleed during follow-up by bleed type and sub-cohort. **eFigure 2**. Adjusted hazard ratios for ethnicity, by subgroup and bleed type.

## Data Availability

The datasets analysed during the current study are not publicly available as they are based on patient data collected as part of health service delivery (i.e. secondary use of patient data) and were provided to the research team under the approval of the New Zealand National Multi Region Ethics Committee (MEC07/19/EXP).

## Abbreviations

CVD: Cardiovascular disease
AF: Atrial fibrillation
adjHRs: Adjusted hazards ratios
MELAA: Middle Eastern/Latin American/African
ICD: International Statistical Classification of Diseases and Related Health Problems
NZDep: New Zealand Index of Deprivation
SBP: Systolic blood pressure
TC:HDL: Total cholesterol to high density lipoprotein cholesterol ratio
IQR: Interquartile ranges
