# Supplementary material for: Risk of major bleeding by ethnicity and socioeconomic deprivation among 488,107 people in primary care: a cohort study
Source: BMC Cardiovasc Disord. 2021 Apr 23;21:206. doi: 10.1186/s12872-021-01993-9 (PMC8063422; doi:10.1186/s12872-021-01993-9)
Supplement: Supplementary file 1 — Additional file1: Table S1. Definitions of variables. Table S2. ICD codes used to identify medical history or outcomes from hospital or death records. Table S3. Medications included in drug classes. Table S4. Adjusted hazard ratios for any bleed, by subgroup. Table S5. Adjusted hazard ratios for gastrointestinal bleed. Table S6. Adjusted hazard ratios for intracranial bleed. Table S7. Adjusted hazard ratios for other bleed. eFigure 1. Cumulative incidence of a bleed during follow-up by bleed type and sub-cohort. eFigure 2. Adjusted hazard ratios for ethnicity, by subgroup and bleed type. [file 12872_2021_1993_MOESM1_ESM.docx]

**eTable 1.** Definitions of variables

| **Variable** | **Source** | **Definition** |
| --- | --- | --- |
| *Demography* | | |
| Sex | National Health Index database | **Female**, male |
| Age | National Health Index database | Age (continuous) at index PREDICT assessment |
| Ethnicity | National Health Index database | Self-reported ethnicity was categorised using the prioritised output method according to national ethnicity data protocols (https://www.health.govt.nz/system/files/documents/publications/ethnicitydataprotocols.pdf), with the South Asian population (of whom 90% are Indian in New Zealand) separated out from the Asian category as they are known to have an elevated risk of CVD: Māori > Pacific > Indian > Chinese > Other Asian > **European** > MELAA (Middle Eastern/Latin American/African) > Other. People with ethnicity in the last 2 categories (MELAA, Other) were excluded from the analysis due to small numbers |
| Socio-economic deprivation | National Health Index database | We used the New Zealand Index of Deprivation (NZDep) (2006) as a measure of socioeconomic position. The NZDep was constructed from 9 census derived variables representing 8 dimensions of deprivation. In this study, deprivation quintiles (1=least deprived, 5=most deprived) rather than the conventional NZDep2006 deciles were used, i.e.  **Deprivation quintile 1 (least deprived)** = NZ Dep decile 1 or 2  Deprivation quintile 2 = NZ Dep decile 3 or 4  Deprivation quintile 3 = NZ Dep decile 5 or 6  Deprivation quintile 4 = NZ Dep decile 7 or 8  Deprivation quintile 5 (most deprived) = NZ Dep decile 9 or 10 |
| *Measurements* | | |
| Systolic blood pressure, mm Hg | PREDICT | **<140 mm Hg**, >/= 140 mm Hg  Mean of two systolic blood pressure measurements obtained at index assessment |
| Ratio of total cholesterol to high density lipoprotein cholesterol, mmol/L | PREDICT | One measure, fasting or non-fasting (continuous) |
| *Medical History* | | |
| Smoking | PREDICT | Smoker = current smoker or ex-smoker who quit less than 12 months prior to index assessment  Ex-smoker = quit 12 months or more prior to index assessment  **Never smoker** = never smoker at index assessment |
| Diabetes | Multiple | **No**, yes; yes if:  - History of diabetes (PREDICT)  AND/OR  - Prior hospitalisation in which diabetes or associated condition noted (ICD-10-AM E10-14 or ICD-9-CM-A 250)  AND/OR  - 1+ dispensing of diabetes medication (see eTable 3 for medications included in class) in the last 6 months |
| Coronary heart disease | Multiple | **No**, yes; yes if:  -History of angina OR myocardial infarction or ischaemic heart disease or percutaneous transluminal coronary angioplasty or coronary artery bypass graft (PREDICT)  AND/OR  -Prior hospitalisation in which atherosclerotic coronary heart disease diagnosis (incl angina) or procedure noted  AND/OR  -Dispensing of 1+ anti-anginal on 3+ occasions in the last 5 years  Relevant ICD codes used to identify relevant hospitalisations (principal and secondary diagnoses considered) and procedures in eTable 2  Medications included within each drug class in eTable 3 |
| Cerebro-vascular disease | Multiple | **No**, yes; yes if:  -History of stroke or transient ischaemic attack (PREDICT)  AND/OR  -Prior hospitalisation in which atherosclerotic cerebrovascular diagnosis (incl ischaemic stroke and transient ischaemic attack) noted    Relevant ICD codes used to identify relevant hospitalisations (principal and secondary diagnoses considered) in eTable 2 |
| Peripheral vascular disease | Multiple | **No**, yes; yes if:  -History of peripheral vascular disease (PREDICT)  AND/OR  -Prior hospitalisation in which atherosclerotic peripheral vascular disease diagnosis or procedures noted  Relevant ICD codes used to identify relevant hospitalisations (principal and secondary diagnoses considered) and procedures in eTable 2 |
| Heart failure | PREDICT | **No**, yes; yes if:  -Prior hospitalisation in which heart failure diagnosis noted (any of ICD-10-AM I50, I110, I130, I132)  AND/OR  -Dispensing of 1+ loop diuretic (frusemide or bumetanide) on 3+ occasions in the last 5 years  AND/OR  - Any dispensing of metolazone in the last 6 months |
| Atrial fibrillation | Multiple | **No**, yes; yes if:  -History of atrial fibrillation from PREDICT  AND/OR  -Prior hospitalisation in which atrial fibrillation diagnosis noted (ICD-10-AM I48) |
| Cancer | NZ Cancer Registry and National Minimum DataSet | **No**, yes; yes if:  Included in NZ Cancer Registry (NZCR) prior to index assessment. NZCR is a population-based register of all primary malignant diseases diagnosed in NZ, excluding squamous and basal cell skin cancers. Reporting is a legislative requirement. Sources of data: laboratories, hospitals and mortality collection. Data from the NZCR were only available up until the end of 2014, therefore cancer history was supplemented with hospitalisation data. People were classified as having a history of cancer if they had a hospitalisation prior to the index assessment in which a relevant cancer ICD code was listed. Relevant cancer ICD codes were those listed in the MoH ICD code list with eligible cancer status = A (always registerable). This ICD code list is used to assist in identifying people potentially eligible for the NZCR. |
| Gastro-intesinal bleed | National Minimum DataSet | **No**, yes; yes if:  Hospitalisation prior to index PREDICT assessment in which the principal or a secondary diagnosis is a gastrointestinal bleed code (see eTable 2 for ICD codes) |
| Other bleed | National Minimum DataSet | **No**, yes; yes if:  Hospitalisation prior to index PREDICT assessment in which the principal or a secondary diagnosis is an other bleed code (see eTable 2 for ICD codes) |
| Peptic ulcer disease | National Minimum DataSet and Pharms database | **No**, yes; yes if:  - Prior hospitalisation in which the principal or a secondary diagnosis is a peptic ulcer disease code (see eTable 2 for ICD codes)  AND/OR  - 1+ dispensing of Helicobacter pylori eradication medication in the last 5 years (see eTable 3 for medications included in class)  AND/OR  - 1+ dispensing of proton pump inhibitor or histamine H2-receptor antagonist in the preceding 6 months (see eTable 3 for medications included in the class) |
| Thrombo-cytopaenia | Testsafe | **No**, yes; yes if:  Most recent platelet count obtained from TestSafe, up to 5 years prior to the index PREDICT assessment, <150 x10^9^/L.  No if most recent platelet count obtained from TestSafe, up to 5 years prior to the index PREDICT assessment, >/=150 x10^9^/L or if no platelet count available. |
| Anaemia |  |  |
| Chronic kidney disease | Multiple | **No**, yes; yes if:  -overt diabetic or other nephropathy (albumin:creatinine 30 mg/mmol OR urinary albumin 200 mg/L) (PREDICT)  AND/OR  -chronic kidney according to their estimated glomerular filtration rates (eGFR) calculated from serum creatinine values obtained from TESTSAFE up to 5 years prior to the PREDICT index assessment  People were categorised as having chronic kidney disease according to their eGFR if they met BOTH of the following criteria  -eGFR <30 using creatinine nearest to index PREDICT assessment, AND  -one other eGFR <30 using creatinine >3 months (90 days) prior to the creatinine nearest to the index PREDICT assessment  The time difference between measures (>3 months) was selected for consistency with the KDIGO definition of chronic kidney disease (KDIGO 2012 clinical practice guideline for the evaluation and management of chronic kidney disease. Kidney Int Suppl 2013;**3**(1))  Notes:  1. Relevant international consensus (KDIGO) eGFR categories (endorsed in NZ. Ministry of Health. Managing chronic kidney disease in primary care: National consensus statement. Wellington: Ministry of Health 2015) are:  - G5 – kidney failure [eGFR <15]  - G4 – severely decreased eGFR [15-29].  2. eGFR calculated using the CKD-EPI equation (as recommended by the Australasian Creatinine Consensus Working Group. Johnson DW, Jones GRD, Mathew TH, et al. Chronic kidney disease and automatic reporting of estimated glomerular filtration rate: new developments and revised recommendations. Med J Aust 2013;**197**:224-5). |
| Chronic liver disease | National Minimum DataSet | **No**, yes; yes if:  Hospitalisation prior to index PREDICT assessment in which the principal or a secondary diagnosis is a chronic liver disease code (see eTable 2 for ICD codes) |
| Chronic pancreatitis | National Minimum DataSet | **No**, yes; yes if:  Hospitalisation prior to index PREDICT assessment in which the principal or a secondary diagnosis is a chronic pancreatitis disease code (see eTable 2 for ICD codes) |
| Chronic alcohol-related disease | National Minimum DataSet | **No**, yes; yes if:  Hospitalisation prior to index PREDICT assessment in which the principal or a secondary diagnosis is a chronic alcohol-related disease code (see eTable 2 for ICD codes) |
| *Medication* | | |
| Antiplatelet | Pharms database | 1+ dispensing in the 6 months prior to index PREDICT risk assessment. See eTable 3 for medications included in class. |
| Anti-coagulant |  |  |
| Anti-hypertensive |  |  |
| Lipid lowering |  |  |
| Non-steroidal anti-inflammatory |  |  |
| Steroid |  |  |
| Selective serotonin re-uptake inhibitor |  |  |

ICD=International Classification of Diseases, NHI=National Health Index, NMDS=National Minimum Dataset

**eTable 2.** ICD codes used to identify medical history or outcomes from hospital or death records

| **Category** | **ICD-10-AM* codes (ICD-9-CM-A codes where applicable)** |
| --- | --- |
| Intracranial bleed | Subarachnoid haemorrhage: I60^‡^(430), Intracerebral haemorrhage: I61^‡^ (431), Other non-traumatic intracranial haemorrhage: I62^‡^ (4320, 4321, 4329), Sequelae of subarachnoid haemorrhage: I690^§^, Sequelae of intracerebral haemorrhage: I691^§^, Sequelae of other intracranial haemorrhage: I692^§^ |
| Coronary artery disease^†^ | Cardiac arrest: I46^‡^  Angina pectoris: I20^‡^, Acute MI: I21^‡^ Subsequent MI: I22^‡^, Complications of acute MI: I23^‡^, Other IHD: I24^‡^ (except I241 – Dressler’s syndrome), Chronic IHD: I25^‡^  Angioplasty/stent(s): 3530400-3530401, 3530500-3530501, 3530906-3530909, 3531000-3531005, Bypass: 3849700-3849707, 3850000-3850004, 3850300-3850304, 9020100-9020103, Other coronary procedures: 3845619, 3850500, 3850700, 3850800, 3850900, 3863700, Presence of coronary procedure: Z951, Z955, Z958, Z959 |
| Cerebro-vascular disease^†^ | Cerebral infarction: I63^‡^, Stroke, not specified as haemorrhage or infarction (as these are usually ischaemic): I64 (no subcategories), Sequelae of cerebral infarction: I693, Sequelae of stroke, not specified as haemorrhage or infarction: I694  Transient ischaemic attack: G45^‡^ (except G454 – transient global amnesia), G46^‡^  Occlusion and stenosis of precerebral arteries, not resulting in cerebral infarction: I65^‡^,Occlusion and stenosis of cerebral arteries, not resulting in cerebral infarction: I66^‡^, Dissection of cerebral arteries, nonruptured: I670, Cerebral atherosclerosis: I672, Sequelae of other and unspecified cerebrovascular disease: I698 |
| Peripheral vascular disease^†^ | Atherosclerosis with symptoms: I702^‡^, Atherosclerosis (other): I700, I701, I7020, I708, I709, Aortic aneurysm and dissection: I71^‡^, Peripheral vascular disease, unspecified: I739, Arterial embolism and thrombosis: I74^b^, Diabetes mellitus with peripheral /other circulatory complications: E105^‡^, E115^‡^, E145^‡^  The following procedures: aneurysm excisions, repairs and replacements, bypasses, endarterectomies and patch grafts, resections and re-anastomoses  Involving the following arteries:  carotid: 327000-3271011, 3270300, 3310000, 3350000  aorta: 3270800-3270803, 3311200, 3311500, 3311800, 3312100, 3315100, 3315400, 3315700, 3316000, 3350900, 3351200, 3351500  femoral: 3271200-3271201, 3271500-3271503, 3271800-3271801, 3273900, 3274200, 3274500, 3274800, 3275100-3275103, 3275400-3275402, 3275700-3275701, 3351501, 3352100, 3354200  mesenteric : 3273000-3273001, 3273300-3273301, 3273600, 3353001, 3353300, 3353600  other: 3276300-3276303, 3276305-3276314, 3276316-3276319, 3305000, 3305500, 3307500, 3308000, 3312400, 3312700, 3313000, 3316300, 3317800, 3318100, 3350600-3350601, 3351800, 3352400, 3352700, 3353000, 3353900, 3354800-3354803, 3355100, 3355400, 3530306-3530307, 3531200-3531201,3531500-3531501, 9022900, 902300 |
| Gastrointestinal bleed | Peptic ulcer disease with bleed and/or perforation from the following sites: Gastric: K250, K251, K252, K254, K255, K256 (53100, 53101, 53110, 53111, 53120, 53121, 53150, 53151, 53160, 53161, 53140, 53141), Duodenal: K260, K261, K262, K264, K265, K266 (53200, 53201, 53210, 53211, 53220, 53221, 53250, 53251, 53260, 53261, 53240, 53241), Gastrojejunal: K280, K281, K282, K284, K285, K286 (53400, 53401, 53410, 53411, 53420, 53421, 53440, 53441, 53450, 53451, 53460, 53461),  Peptic / unspecified site: K270, K271, K272, K274, K275, K276 (53300, 53301, 53310, 53311, 53320, 53321, 53340, 53341, 53350, 53351, 53360, 53361)  (Diverticulitis or diverticulosis) with bleed: K5703, K5713, K5711, K5721, K5723, K5731, K5733, K5741, K5743 K5751, K5753, K5781, K5783, K5791, K5793 (56202, 56203, 56212, 56213)  Angiodysplasia with bleed: K3182, K5522 (53783, 56985)  Mallory-Weiss tear: K226 (5307)  (Gastritis or gastro-duodenitis or duodenitis) with bleed: K290, K2921^(8th)^, K2931^(8th)^, K2941^(8th)^ (atrophic gastritis with haemorrhage), K2951^(8th)^, K2961^(8th)^, K2981^(8th)^, K2971^(8th)^, K2997^(8th)^ (53501, 53511 (atrophic gastritis with haemorrhage), 53531, 53541, 53551, 53561)  Haemorrhage of anus and rectum: K625 (5693)  Haematemesis: K920 (5780)  Melaena: K921 (5781)  Gastrointestinal haemorrhage, unspecified: K922 (5789)  Oesophageal varices with bleeding: I850, I9821^(1st, 2nd, 3rd)^, I983 ^(6th, 8th)^ (4560, 45620)  Oesophageal haemorrhage: (53082) |
| Other bleed | Ocular (vitreous and retinal): H356, H431 (36281, 37923)  Respiratory passage(inc. epistaxis and haemoptysis): R04 (7847, 7848, 7863)  Haemopericardium / haemoperitoneum: I312, K661 (4230, 56881)  Haemarthrosis: M250^‡^ (71910, 71911, 71912, 71913, 71914, 71915, 71916, 71917, 71918, 71918, 71919) |
| Peptic ulcer disease (non-bleeding / non-perforated) | From the following sites: Oesophagus: K221 (5302), Gastric: K253, K257, K259 (53130, 53131, 53170, 53171, 53190, 53191), Duodenal: K263, K267, K269 (53230, 53231, 53270, 53271, 53290, 53291), Peptic / site unspecified: K273, K277, K279 (53330, 53331, 53370, 53371, 53390, 53391), Gastrojejunal: K283, K287, K289 (53430, 53431, 53470, 53471, 53490, 53491)  History of peptic ulcer disease Z8711 (V1271) |
| Chronic liver disease | Gastro-oesophageal varices: I850, I859, I864 (4560, 4561), Alcoholic chronic liver disease: K702, K703, K704 (5712), Chronic hepatic failure: K721, Other cirrhosis of liver (incl biliary and toxic): K717, K743, K744, K745, K746 (5715, 5716), Portal hypertension: K766 (5723), Hepatorenal syndrome: K767 (5724) |
| Chronic pancreatitis | Chronic pancreatitis: K860, K861 (5771) |
| Chronic alcohol-related condition | Alcohol-induced pseudo-Cushing’s syndrome: E244, Degeneration of nervous system due to alcohol: G312, Alcoholic polyneuropathy: G621 (3575), Alcoholic myopathy: G721, Alcoholic cardiomyopathy: I426 (4255), Alcoholic gastritis: K292^(1st, 2nd, 3rd, 4th)^, K2920^(8th)^, K2921^(8th)^ (53530, 53531), Alcoholic liver disease: K70^‡^ (5710, 5711, 5712, 5713), Alcohol-induced chronic pancreatitis K860, Mental and behavioural disorders due to use of alcohol: F10^‡^, except acute intoxication – F100 and harmful use – F101 (2910, 2911, 2912, 2913, 2915, 2918, 2919, 30390, 30391, 30392, 30393), History of alcohol use disorder Z8641 (V1584), Alcohol counselling, detoxification or rehabilitation: Z502, Z714, 9201000, 9200200, 9200300, 9200400, 9200800, 9200900 |

Hospital records from 1 January 1988 to 31 December 2017

ICD-9-CM-A= Australian Version of The International Classification of Diseases, 9th Revision, Clinical Modification, ICD-10-AM=International Statistical Classification of Diseases and Related Health Problems, Australian Modification

^*^Same codes used for all ICD-10-AM editions used in New Zealand to date (ie 1^st^, 2^nd^, 3^rd^, 6^th^ and 8^th^) unless otherwise specified

^†^These are the codes used by the Vascular Informatics Using Epidemiology and the Web (VIEW) team, Department of Epidemiology and Biostatistics, University of Auckland (at March 2016) to identify people with CVD from hospital records. Only ICD-10-AM codes were used because diagnoses and procedures were mapped by the Ministry of Health to ICD-10-AM 2nd edition (where mappings existed), as well as the original submitted ICD-9-CM-A /ICD-10-AM version

^‡^Includes any subcategories that come after the last number, unless specified as excluded

^§^Used only to identify people with history of bleed (i.e. not for bleeding outcomes)

**eTable 3.** Medications Included in Drug Classes

| **Increase bleeding risk** | | | | **Peptic ulcer disease medication** | | | **Treat other diseases** | |
| --- | --- | --- | --- | --- | --- | --- | --- | --- |
| **Antiplatelet**  Aspirin (irrespective of dosage, excluding combinations for cold & ‘flu)  Clopidogrel  Dipyridamole  Prasugrel  Ticagrelor  Ticlopidine  **Anticoagulant**  Dabigatran  Phenindione  Rivaroxaban  Warfarin  **Steroid**  Betamethasone  Cortisone  Dexamethasone  Fludrocortisone  Hydrocortisone  Methylprednisolone  Prednisolone  Prednisone | | **Non-steroidal anti-inflammatory**  Diclofenac  Diflunisal  Fenbufen  Fenoprofen  Flubriprofen  Ibuprofen  Indomethacin  Ketoprofen  Mefenamic acid  Naproxen  Pheylbutazone  Prixoicam  Suldinac  Tenoxicam  Tiaprofenic acid  **Selective serotonin re-uptake inhibitor**  Citalopram  Escitalopram  Fluoxetine  Nefazodone  Paroxetine  Sertraline | | **Proton pump inhibitor or histamine H2-receptor antagonist**  Lansoprazole  Omeprazole  Pantoprazole  Ranitidine  **Helicobacter pylori eradication**  Clarithromycin 500mg  Combination of:  -Bismuth  -Metronidazole  -Tetracycline  Combination of:  -Omeprazole  -Amoxicillin  -Clarithromycin or metronidazole | | | **Heart failure**  Bumetanide  Frusemide  Metolazone  **Antianginal**  Glyceryl trinitrate  Isosorbide dinitrate / mononitrate  Nicorandil  Pentaerythritol tetranitrate  Perhexiline maleate  **Diabetes**  Insulin  Acarbose  Chlorpropramide  Glibenclamide  Gliclazide  Glipizide  Metformin  Pioglitazone  Rosiglitazone  Tolazamide  Tolbutamide | |
| **Blood pressure-lowering*** | | | | | | | | **Lipid- lowering** |
| **Angiotensin-converting enzyme inhibitor**  Benazepril  Captopril  Cilazapril  Enalapril  Lisinopril  Perindopril  Quinapril  Trandolapril | **Angiotensin II receptor blocker**  Candesartan  Losartan  **Beta blocker**  Acebutolol  Alprenolol  Atenolol  Bisoprolol  Carvedilol | | **Beta blocker**  Celiprolol  Labetalol  Metoprolol  Nadolol  Oxprenolol  Pindolol  Propranolol  Sotalol  Timolol | | **Calcium channel blocker**  Amlodipine  Diltiazem  Felodipine  Isradipine  Nifedipine  Verapamil  **Other**  Amiloride  Clonidine  Clopamide  Hydralazine  Methyldopa  Triamterene | **Thiazide**  Bendro-fluazide  Chlor-thalidone  Chloro-thiazide  Cyclo-penthiazide Hydrochloro-thiazide  Indapamide  Methy-clothiazide | | **Statin**  Atorvastatin  Fluvastatin  Pravastatin  Simvastatin  **Other**  Acipimox  Bezafibrate  Chole-styramine  Clofibrate  Colestipol  Ezetimibe  Gemfibrozil  Nicotinic acid |

Medication dispensing information (Pharmaceutical claims collection database) available from 1 January 2005 to 31 December 2017

Formulations included: oral (tablet, capsule, liquid), patch, suppository, injection (insulin only)

Formulations excluded: cream, ointment, powder, inhaler, injection (except for insulin)

*Alpha blockers, loop diuretics (bumetanide, frusemide), metolazone and spironolactone excluded as the primary indication is not usually to reduce blood pressure

**eTable 4.** Adjusted Hazard Ratios for any bleed, by subgroup

| **Variables**^a^ | **Atrial fibrillation** | **Cardiovascular disease** | **No atrial fibrillation or cardiovascular disease** |
| --- | --- | --- | --- |
| *Demography* | | | |
| Age | 1.02 (1.01-1.03) | 1.03 (1.02-1.04) | 1.04 (1.04-1.04) |
| Male | 0.99 (0.87-1.12) | 1.06 (0.97-1.17) | 1.33 (1.26-1.40) |
| Ethnicity |  |  |  |
| Māori | 1.63 (1.39-1.91) | 1.24 (1.09-1.42) | 1.57 (1.45-1.70) |
| Pacific | 1.90 (1.58-2.28) | 1.30 (1.12-1.51) | 1.62 (1.49-1.75) |
| Indian | 0.75 (0.48-1.19) | 0.98 (0.82-1.19) | 0.95 (0.85-1.06) |
| Chinese | 1.53 (1.08-2.16) | 1.15 (0.90-1.47) | 1.13 (1.01-1.26) |
| Other Asian | 1.22 (0.65-2.29) | 1.05 (0.75-1.46) | 1.34 (1.17-1.52) |
| Socioeconomic deprivation, per quintile | 1.07 (1.02-1.12) | 1.07 (1.03-1.10) | 1.10 (1.08-1.12) |
| *Cardiovascular History* | | | |
| Coronary heart disease | 1.18 (1.04-1.35) | 1.00 (0.90-1.11) | Not applicable |
| Cerebrovascular disease | 1.16 (1.00-1.34) | 1.18 (1.06-1.32) | Not applicable |
| Peripheral vascular disease | 1.27 (1.08-1.48) | 1.23 (1.09-1.37) | Not applicable |
| Heart failure | 1.38 (1.21-1.57) | 1.29 (1.16-1.44) | Not applicable |
| Diabetes | 0.96 (0.84-1.10) | 1.11 (1.01-1.23) | 1.20 (1.12-1.29) |
| Smoker | 1.03 (0.86-1.24) | 1.31 (1.16-1.48) | 1.46 (1.37-1.56) |
| Ex-smoker | 1.09 (0.96-1.25) | 1.02 (0.92-1.14) | 1.14 (1.07-1.22) |
| *Medical History* | | | |
| Systolic BP >/= 140 mm Hg | 1.11 (0.98-1.27) | 1.10 (1.00-1.20) | 1.13 (1.07-1.20) |
| TC:HDL ratio, mmol/L | 1.02 (0.97-1.07) | 0.99 (0.96-1.03) | 1.01 (0.99-1.03) |
| Low haemoglobin | 1.52 (1.30-1.77) | 1.48 (1.31-1.67) | 1.87 (1.71-2.05) |
| Peptic ulcer disease | 1.01 (0.89-1.15) | 1.11 (1.01-1.22) | 1.33 (1.25-1.41) |
| Gastrointestinal bleed | 1.77 (1.51-2.07) | 2.25 (1.99-2.55) | 2.75 (2.50-3.02) |
| Other^d^ bleed | 1.52 (1.27-1.82) | 1.65 (1.38-1.96) | 2.13 (1.82-2.50) |
| Thrombocytopenia | 1.18 (0.95-1.46) | 1.26 (1.04-1.53) | 1.74 (1.52-1.99) |
| Chronic kidney disease | 1.61 (1.31-1.99) | 1.73 (1.47-2.03) | 1.56 (1.30-1.87) |
| Chronic liver disease | 1.45 (0.87-2.43) | 2.01 (1.47-2.76) | 1.86 (1.44-2.40) |
| Chronic pancreatitis | 1.54 (0.63-3.75) | 1.36 (0.72-2.57) | 0.88 (0.51-1.50) |
| Alcohol-related disease | 1.32 (0.99-1.76) | 1.50 (1.19-1.90) | 2.02 (1.71-2.39) |
| Cancer | 1.33 (1.14-1.56) | 1.35 (1.19-1.52) | 1.46 (1.34-1.58) |
| *Medication History* | | | |
| Antiplatelet | 1.10 (0.96-1.26) | 1.13 (1.01-1.27) | 1.27 (1.18-1.37) |
| Non-aspirin antiplatelet | 0.83 (0.69-1.00) | 1.16 (1.04-1.30) | 1.15 (1.08-1.22) |
| Blood pressure-lowering | 1.31 (1.06-1.61) | 1.12 (0.99-1.27) | 1.25 (1.18-1.33) |
| Anticoagulant | 1.53 (1.34-1.75) | 1.78 (1.45-2.18) | 2.23 (1.74-2.86) |
| Lipid-lowering | 0.98 (0.85-1.12) | 0.97 (0.86-1.09) | 0.95 (0.89-1.02) |
| Steroid | 1.11 (0.94-1.30) | 1.26 (1.12-1.43) | 1.37 (1.26-1.49) |
| Selective serotonin re-uptake inhibitors | 1.14 (0.91-1.42) | 1.11 (0.96-1.30) | 1.24 (1.12-1.37) |

BP = blood pressure, HDL = high density lipoprotein cholesterol, TC=total cholesterol

All of the variables were included in each of the models (except those listed as not applicable).

The total number of people included (and excluded due to a missing value) in the models for atrial fibrillation, cardiovascular disease and no atrial fibrillation or cardiovascular disease were: 15097 (115), 43437 (353) and 425668 (3437), respectively.

For the atrial fibrillation subgroup, the number of bleeds in the models were: any (1202), gastrointestinal (723), intracranial (172), other (379). For the cardiovascular disease subgroup, the number of bleeds in the models were: any (2092), gastrointestinal (1470), intracranial (270), other (423). For the no atrial fibrillation or cardiovascular disease subgroup, the number of bleeds in the models were: any (6478), gastrointestinal (4482), intracranial (926), other (1196).

**eTable 5.** Adjusted Hazard Ratio for Gastrointestinal Bleed

| **Variables**^a^ | **Atrial fibrillation** | **Cardiovascular disease** | **No atrial fibrillation or cardiovascular disease** |
| --- | --- | --- | --- |
| *Demography* | | | |
| Age | 1.03 (1.02-1.04) | 1.03 (1.03-1.04) | 1.04 (1.04-1.04) |
| Male | 1.04 (0.89-1.22) | 1.11 (0.99-1.24) | 1.39 (1.31-1.48) |
| Self-identified ethnicity |  |  |  |
| Māori | 1.48 (1.21-1.82) | 1.17 (1.00-1.38) | 1.47 (1.34-1.62) |
| Pacific | 1.64 (1.29-2.08) | 1.08 (0.90-1.30) | 1.51 (1.37-1.66) |
| Indian | 0.59 (0.31-1.11) | 0.93 (0.75-1.16) | 0.93 (0.81-1.06) |
| Chinese | 1.43 (0.92-2.22) | 1.06 (0.78-1.43) | 1.03 (0.90-1.17) |
| Other Asian | 1.00 (0.41-2.43) | 0.93 (0.62-1.41) | 1.24 (1.06-1.45) |
| Socioeconomic deprivation, per quintile | 1.07 (1.01-1.13) | 1.09 (1.04-1.13) | 1.11 (1.08-1.13) |
| *Cardiovascular History* | | | |
| Coronary heart disease | 1.23 (1.04-1.46) | 1.08 (0.95-1.22) | Not applicable |
| Cerebrovascular disease | 1.10 (0.90-1.33) | 1.14 (0.99-1.30) | Not applicable |
| Peripheral vascular disease | 1.26 (1.03-1.54) | 1.28 (1.12-1.46) | Not applicable |
| Heart failure | 1.32 (1.12-1.57) | 1.31 (1.16-1.49) | Not applicable |
| Diabetes | 1.11 (0.94-1.32) | 1.03 (0.92-1.16) | 1.20 (1.10-1.30) |
| Smoker | 1.17 (0.93-1.49) | 1.40 (1.21-1.62) | 1.43 (1.33-1.55) |
| Ex-smoker | 1.14 (0.97-1.35) | 1.02 (0.90-1.15) | 1.12 (1.03-1.21) |
| *Medical History* | | | |
| Systolic BP >/= 140 mm Hg | 1.11 (0.94-1.31) | 1.07 (0.96-1.19) | 1.01 (0.95-1.09) |
| TC:HDL ratio, mmol/L | 1.04 (0.98-1.10) | 1.00 (0.95-1.04) | 1.01 (0.99-1.04) |
| Low haemoglobin | 1.61 (1.33-1.95) | 1.52 (1.32-1.75) | 1.98 (1.78-2.20) |
| Peptic ulcer disease | 0.94 (0.79-1.11) | 1.21 (1.09-1.36) | 1.37 (1.27-1.47) |
| Gastrointestinal bleed | 2.41 (2.00-2.90) | 2.64 (2.29-3.04) | 3.39 (3.06-3.77) |
| Other^d^ bleed | 1.06 (0.82-1.37) | 1.14 (0.89-1.45) | 1.44 (1.15-1.81) |
| Thrombocytopenia | 1.35 (1.04-1.75) | 1.24 (0.99-1.56) | 1.85 (1.59-2.16) |
| Chronic kidney disease | 1.77 (1.37-2.29) | 1.82 (1.50-2.21) | 1.41 (1.12-1.77) |
| Chronic liver disease | 1.22 (0.64-2.34) | 2.19 (1.52-3.14) | 1.92 (1.44-2.54) |
| Chronic pancreatitis | 1.77 (0.65-4.78) | 1.37 (0.67-2.79) | 0.94 (0.53-1.69) |
| Alcohol-related disease | 1.41 (0.99-2.00) | 1.43 (1.09-1.88) | 0.94 (0.53-1.69) |
| Cancer | 1.51 (1.25-1.82) | 1.36 (1.18-1.57) | 1.39 (1.26-1.54) |
| *Medication History* | | | |
| Antiplatelet | 1.09 (0.92-1.30) | 1.10 (0.96-1.26) | 1.27 (1.16-1.39) |
| Non-aspirin nonsteroidal anti-inflammatory | 0.79 (0.61-1.01) | 1.25 (1.09-1.42) | 1.20 (1.12-1.29) |
| Blood pressure-lowering | 1.30 (1.00-1.70) | 1.17 (1.00-1.36) | 1.24 (1.15-1.34) |
| Anticoagulant | 1.28 (1.08-1.52) | 1.31 (0.99-1.72) | 1.70 (1.22-2.37) |
| Lipid-lowering | 0.91 (0.76-1.09) | 0.99 (0.86-1.13) | 0.96 (0.88-1.04) |
| Steroid | 1.20 (0.98-1.47) | 1.21 (1.04-1.40) | 1.36 (1.23-1.51) |
| Selective serotonin re-uptake inhibitors | 1.07 (0.80-1.43) | 1.14 (0.96-1.37) | 1.32 (1.17-1.49) |

BP = blood pressure, HDL = high density lipoprotein cholesterol, TC=total cholesterol

All of the variables were included in each of the models (except those listed as not applicable).

The total number of people included (and excluded due to a missing value) in the models for atrial fibrillation, cardiovascular disease and no atrial fibrillation or cardiovascular disease were: 15097 (115), 43437 (353) and 425668 (3437), respectively.

For the atrial fibrillation subgroup, the number of bleeds in the models were: any (1202), gastrointestinal (723), intracranial (172), other (379). For the cardiovascular disease subgroup, the number of bleeds in the models were: any (2092), gastrointestinal (1470), intracranial (270), other (423). For the no atrial fibrillation or cardiovascular disease subgroup, the number of bleeds in the models were: any (6478), gastrointestinal (4482), intracranial (926), other (1196).

**eTable 6.** Adjusted Hazard Ratio for Intracranial Bleed

| **Variables**^a^ | **Atrial fibrillation** | **Cardiovascular disease** | **No atrial fibrillation or cardiovascular disease** |
| --- | --- | --- | --- |
| *Demography* | | | |
| Age | 1.01 (0.99-1.03) | 1.02 (1.01-1.04) | 1.05 (1.05-1.06) |
| Male | 1.03 (0.75-1.42) | 0.96 (0.74-1.24) | 1.20 (1.05-1.37) |
| Self-identified ethnicity |  |  |  |
| Māori | 1.49 (0.97-2.30) | 0.96 (0.63-1.47) | 1.44 (1.16-1.78) |
| Pacific | 1.97 (1.23-3.16) | 2.09 (1.43-3.05) | 1.73 (1.40-2.14) |
| Indian | 1.00 (0.36-2.79) | 1.67 (1.05-2.64) | 1.38 (1.05-1.82) |
| Chinese | 3.51 (1.79-6.87) | 1.77 (0.99-3.16) | 1.45 (1.12-1.89) |
| Other Asian | 2.60 (0.81-8.32) | 2.08 (1.01-4.29) | 1.67 (1.21-2.30) |
| Socioeconomic deprivation, per quintile | 1.23 (1.09-1.40) | 1.00 (0.91-1.10) | 1.07 (1.02-1.12) |
| *Cardiovascular History* | | | |
| Coronary heart disease | 0.87 (0.62-1.23) | 0.80 (0.60-1.08) | Not applicable |
| Cerebrovascular disease | 2.09 (1.48-2.95) | 1.60 (1.18-2.16) | Not applicable |
| Peripheral vascular disease | 1.31 (0.86-1.98) | 1.02 (0.73-1.43) | Not applicable |
| Heart failure | 1.80 (1.27-2.54) | 1.20 (0.88-1.63) | Not applicable |
| Diabetes | 1.02 (0.71-1.45) | 1.42 (1.09-1.86) | 1.11 (0.92-1.35) |
| Smoker | 1.20 (0.77-1.86) | 1.27 (0.90-1.80) | 1.81 (1.53-2.15) |
| ex-Smoker | 0.75 (0.51-1.09) | 1.01 (0.74-1.36) | 1.10 (0.91-1.32) |
| *Medical History* | | | |
| Systolic BP >/= 140 mm Hg | 1.48 (1.07-2.06) | 1.33 (1.04-1.72) | 1.59 (1.39-1.83) |
| TC:HDL ratio, mmol/L | 0.93 (0.81-1.07) | 0.95 (0.86-1.06) | 0.99 (0.94-1.05) |
| Low haemoglobin | 1.35 (0.89-2.03) | 1.52 (1.09-2.13) | 1.51 (1.16-1.97) |
| Peptic ulcer disease | 1.41 (1.01-1.96) | 0.78 (0.59-1.03) | 1.17 (0.98-1.39) |
| Gastrointestinal bleed | 0.81 (0.49-1.33) | 1.56 (1.04-2.33) | 1.64 (1.20-2.24) |
| Other^d^ bleed | 1.30 (0.79-2.15) | 1.44 (0.86-2.42) | 2.66 (1.79-3.94) |
| Thrombocytopenia | 1.38 (0.80-2.37) | 1.58 (0.97-2.59) | 1.36 (0.91-2.02) |
| Chronic kidney disease | 1.75 (1.02-3.00) | 1.25 (0.78-2.01) | 1.67 (1.01-2.76) |
| Chronic liver disease | 1.60 (0.38-6.75) | 1.98 (0.87-4.50) | 1.78 (0.80-3.96) |
| Chronic pancreatitis | 0.00 (0.00-Inf) | 0.85 (0.12-6.25) | 0.61 (0.08-4.46) |
| Alcohol-related disease | 0.97 (0.42-2.26) | 2.70 (1.54-4.73) | 1.90 (1.15-3.14) |
| Cancer | 0.98 (0.61-1.58) | 1.66 (1.19-2.32) | 1.55 (1.25-1.92) |
| *Medication History* | | | |
| Antiplatelet | 1.00 (0.70-1.42) | 1.26 (0.92-1.74) | 1.18 (0.97-1.44) |
| Non-aspirin nonsteroidal anti-inflammatory | 0.81 (0.49-1.35) | 0.91 (0.64-1.27) | 0.92 (0.78-1.10) |
| BP-lowering | 0.72 (0.45-1.15) | 1.02 (0.72-1.43) | 1.19 (1.02-1.40) |
| Anticoagulant | 1.59 (1.11-2.28) | 3.24 (2.04-5.16) | 1.85 (0.87-3.90) |
| Lipid-lowering | 0.99 (0.68-1.42) | 0.89 (0.65-1.22) | 0.86 (0.72-1.04) |
| Steroid | 0.84 (0.53-1.35) | 1.08 (0.74-1.57) | 1.21 (0.95-1.55) |
| Selective serotonin re-uptake inhibitor | 1.45 (0.83-2.53) | 1.44 (0.96-2.16) | 1.03 (0.76-1.39) |

BP = blood pressure, HDL = high density lipoprotein cholesterol, TC=total cholesterol

All of the variables were included in each of the models (except those listed as not applicable).

The total number of people included (and excluded due to a missing value) in the models for atrial fibrillation, cardiovascular disease and no atrial fibrillation or cardiovascular disease were: 15097 (115), 43437 (353) and 425668 (3437), respectively.

For the atrial fibrillation subgroup, the number of bleeds in the models were: any (1202), gastrointestinal (723), intracranial (172), other (379). For the cardiovascular disease subgroup, the number of bleeds in the models were: any (2092), gastrointestinal (1470), intracranial (270), other (423). For the no atrial fibrillation or cardiovascular disease subgroup, the number of bleeds in the models were: any (6478), gastrointestinal (4482), intracranial (926), other (1196).

**eTable 7.** Adjusted Hazard Ratio for Other Bleed

| **Variables**^a^ | **Atrial fibrillation** | **Cardiovascular disease** | **No atrial fibrillation or cardiovascular disease** |
| --- | --- | --- | --- |
| *Demography* | | | |
| Age | 1.01 (1.00-1.02) | 1.02 (1.01-1.03) | 1.04 (1.03-1.04) |
| Male | 0.89 (0.72-1.10) | 0.96 (0.78-1.17) | 1.23 (1.09-1.38) |
| Self-identified ethnicity |  |  |  |
| Māori | 1.98 (1.50-2.61) | 1.52 (1.15-2.03) | 2.28 (1.92-2.70) |
| Pacific | 2.35 (1.71-3.24) | 1.65 (1.22-2.24) | 2.05 (1.71-2.46) |
| Indian | 0.94 (0.44-2.04) | 0.86 (0.55-1.34) | 0.73 (0.54-0.98) |
| Chinese | 0.75 (0.31-1.82) | 1.17 (0.67-2.07) | 1.33 (1.03-1.71) |
| Other Asian | 0.78 (0.19-3.17) | 0.62 (0.23-1.68) | 1.47 (1.09-1.99) |
| Socioeconomic deprivation, per quintile | 1.01 (0.93-1.09) | 1.04 (0.96-1.12) | 1.08 (1.03-1.13) |
| *Cardiovascular History* | | | |
| Coronary heart disease | 1.24 (0.98-1.56) | 0.88 (0.70-1.10) | Not applicable |
| Cerebrovascular disease | 1.07 (0.82-1.39) | 1.04 (0.80-1.34) | Not applicable |
| Peripheral vascular disease | 1.18 (0.89-1.57) | 1.09 (0.84-1.41) | Not applicable |
| Heart failure | 1.43 (1.13-1.80) | 1.31 (1.04-1.67) | Not applicable |
| Diabetes | 0.69 (0.54-0.89) | 1.27 (1.02-1.58) | 1.31 (1.11-1.54) |
| Smoker | 0.65 (0.44-0.94) | 1.04 (0.78-1.38) | 1.32 (1.13-1.54) |
| Ex-smoker | 1.05 (0.83-1.32) | 1.10 (0.87-1.38) | 1.22 (1.05-1.42) |
| *Medical History* | | | |
| Systolic BP >/= 140 mm Hg | 1.08 (0.86-1.37) | 1.10 (0.90-1.34) | 1.30 (1.15-1.48) |
| TC:HDL ratio, mmol/L | 0.99 (0.91-1.09) | 1.00 (0.92-1.09) | 1.02 (0.98-1.07) |
| Low haemoglobin | 1.45 (1.11-1.91) | 1.44 (1.11-1.88) | 1.97 (1.61-2.41) |
| Peptic ulcer disease | 0.93 (0.74-1.18) | 0.97 (0.79-1.21) | 1.32 (1.14-1.53) |
| Gastrointestinal bleed | 1.19 (0.87-1.61) | 1.47 (1.08-2.00) | 1.21 (0.90-1.62) |
| Other^d^ bleed | 2.89 (2.23-3.75) | 3.54 (2.66-4.71) | 5.02 (3.93-6.40) |
| Thrombocytopenia | 1.31 (0.90-1.90) | 1.12 (0.72-1.76) | 1.74 (1.27-2.40) |
| Chronic kidney disease | 1.77 (1.22-2.56) | 2.17 (1.59-2.96) | 2.11 (1.51-2.95) |
| Chronic liver disease | 1.67 (0.67-4.17) | 1.81 (0.87-3.77) | 1.11 (0.50-2.45) |
| Chronic pancreatitis | 1.49 (0.21-10.76) | 1.80 (0.44-7.41) | 0.45 (0.06-3.24) |
| Alcohol-related disease | 1.30 (0.76-2.23) | 1.40 (0.80-2.46) | 1.77 (1.15-2.75) |
| Cancer | 1.12 (0.83-1.51) | 1.31 (0.99-1.74) | 1.69 (1.40-2.03) |
| *Medication History* | | | |
| Antiplatelet | 1.13 (0.89-1.43) | 1.13 (0.88-1.45) | 1.29 (1.09-1.52) |
| Non-aspirin non-steroidal anti-inflammatory | 0.87 (0.62-1.23) | 1.00 (0.77-1.29) | 1.12 (0.98-1.29) |
| BP-lowering | 1.76 (1.15-2.68) | 1.04 (0.78-1.38) | 1.34 (1.16-1.55) |
| Anticoagulant | 2.09 (1.64-2.68) | 2.76 (1.90-4.00) | 4.30 (2.85-6.49) |
| Lipid-lowering | 0.95 (0.75-1.21) | 1.05 (0.81-1.36) | 0.97 (0.83-1.13) |
| Steroid | 1.03 (0.77-1.37) | 1.46 (1.13-1.89) | 1.62 (1.35-1.95) |
| Selective serotonin re-uptake inhibitor | 1.18 (0.79-1.78) | 0.83 (0.56-1.23) | 1.11 (0.86-1.43) |

BP = blood pressure, HDL = high density lipoprotein cholesterol, TC=total cholesterol

All of the variables were included in each of the models (except those listed as not applicable).

The total number of people included (and excluded due to a missing value) in the models for atrial fibrillation, cardiovascular disease and no atrial fibrillation or cardiovascular disease were: 15097 (115), 43437 (353) and 425668 (3437), respectively.

For the atrial fibrillation subgroup, the number of bleeds in the models were: any (1202), gastrointestinal (723), intracranial (172), other (379). For the cardiovascular disease subgroup, the number of bleeds in the models were: any (2092), gastrointestinal (1470), intracranial (270), other (423). For the no atrial fibrillation or cardiovascular disease subgroup, the number of bleeds in the models were: any (6478), gastrointestinal (4482), intracranial (926), other (1196).

**eFigure 1.** Cumulative incidence of a bleed during follow-up by bleed type and sub-cohort


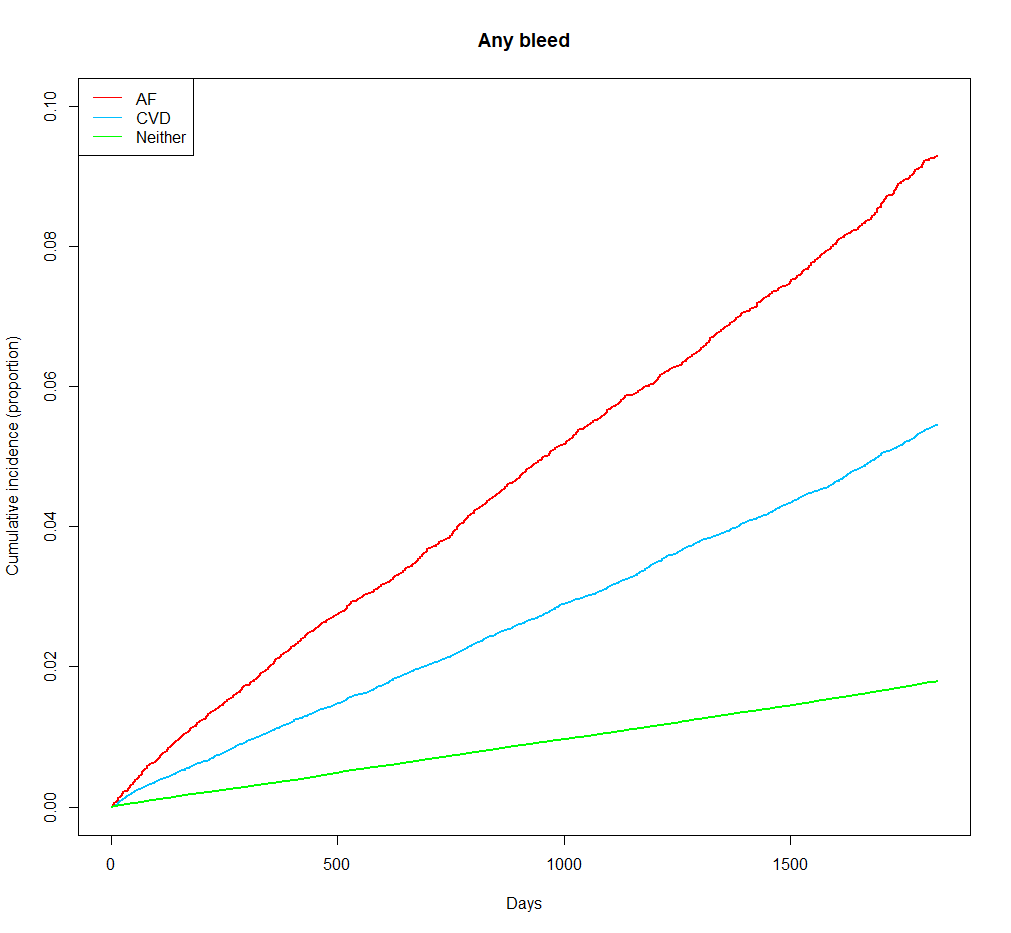

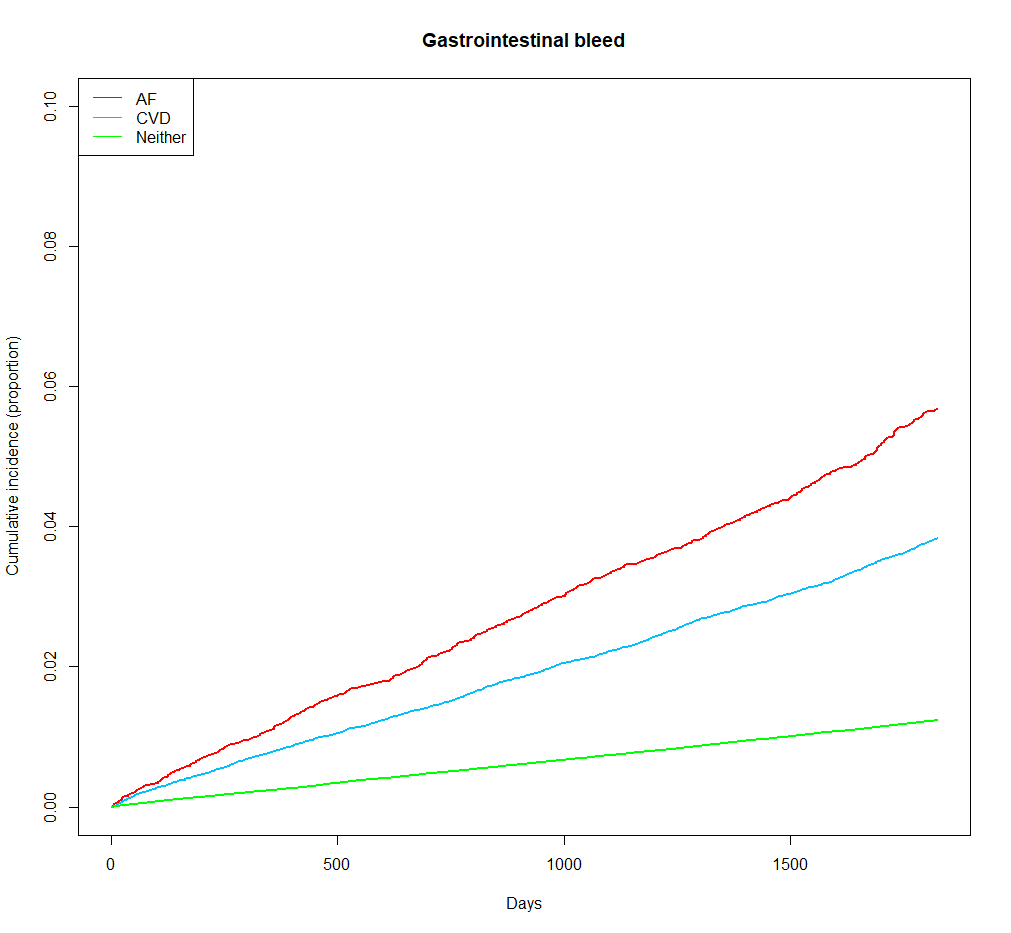


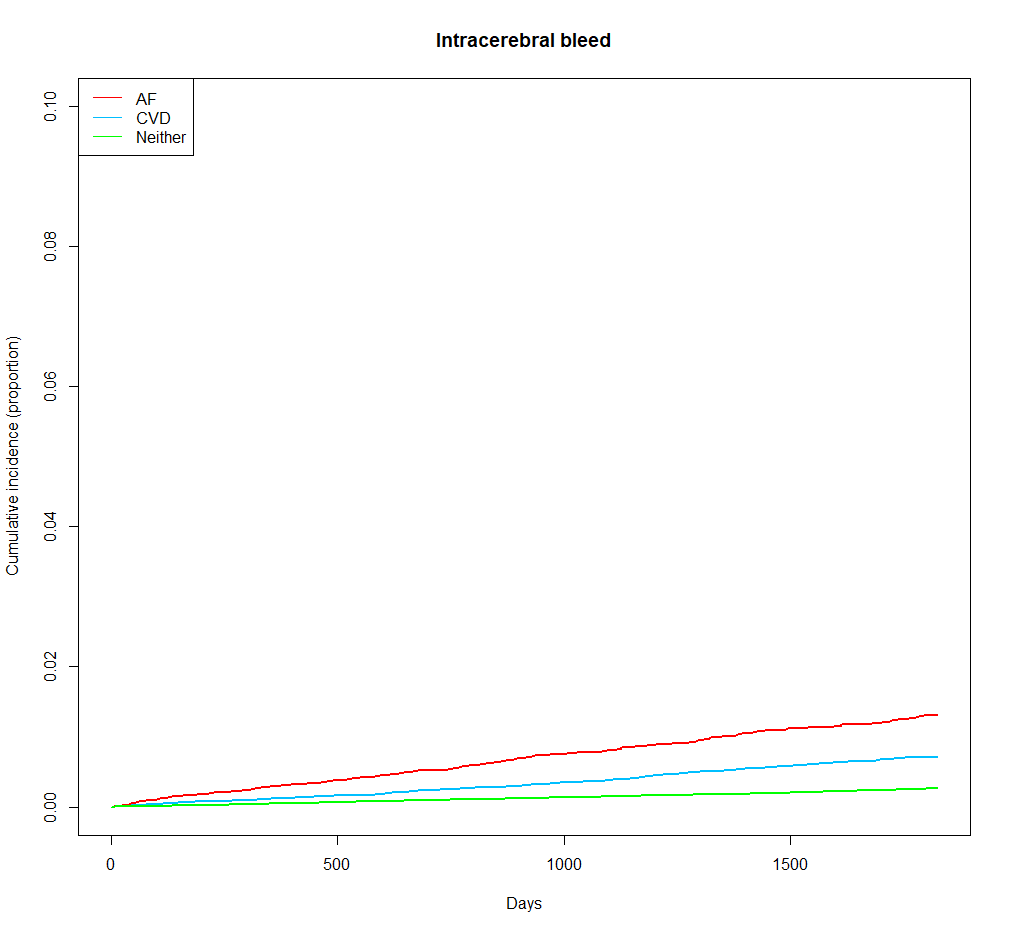

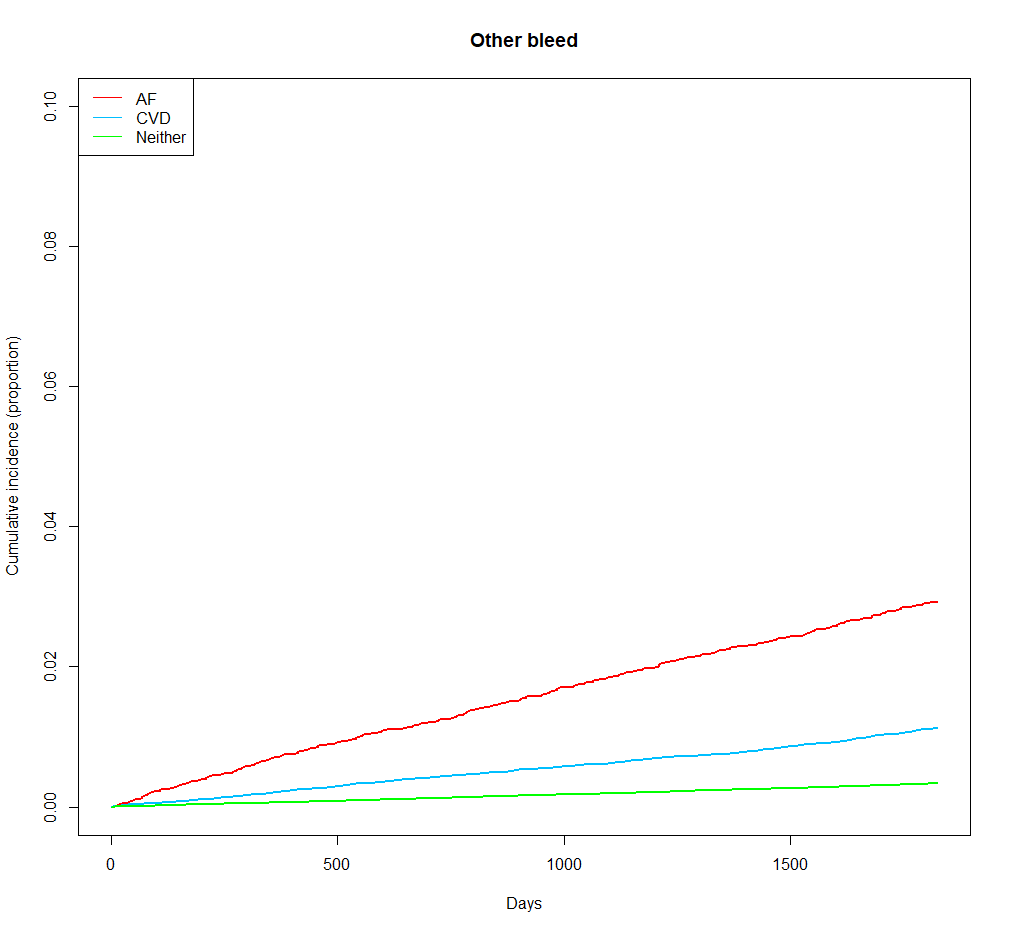


**eFigure 2.** Adjusted hazard ratios for ethnicity, by subgroup and bleed type


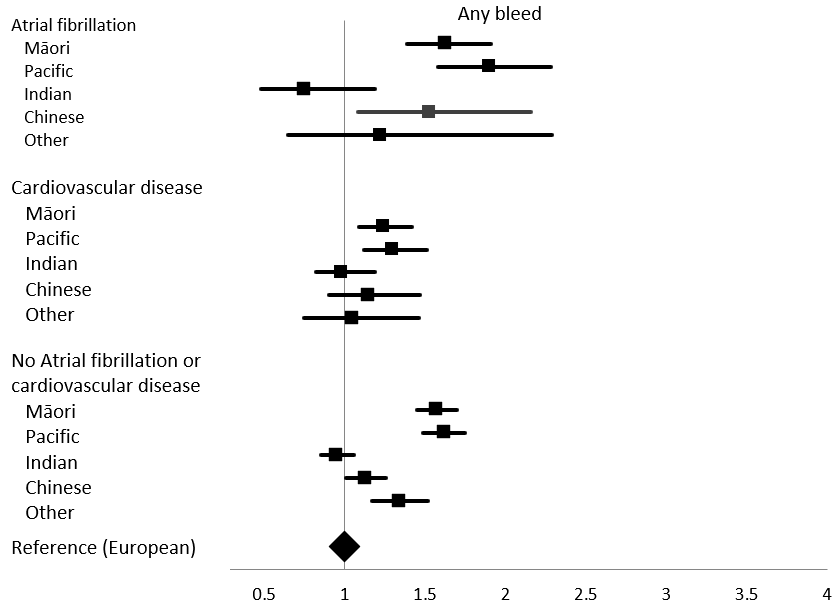

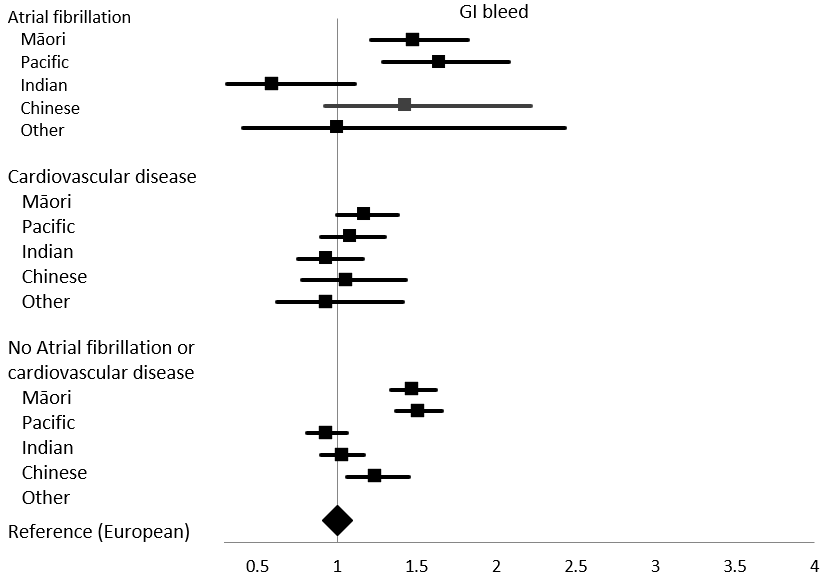


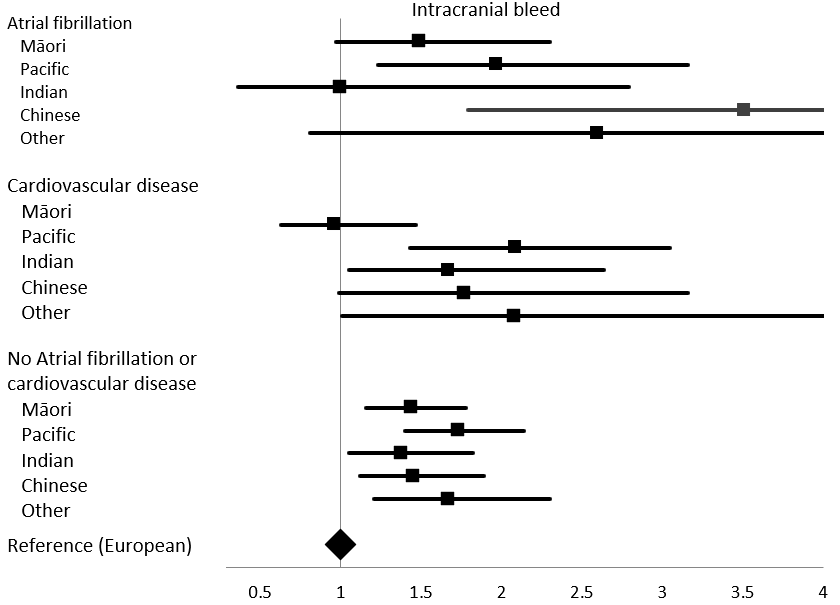

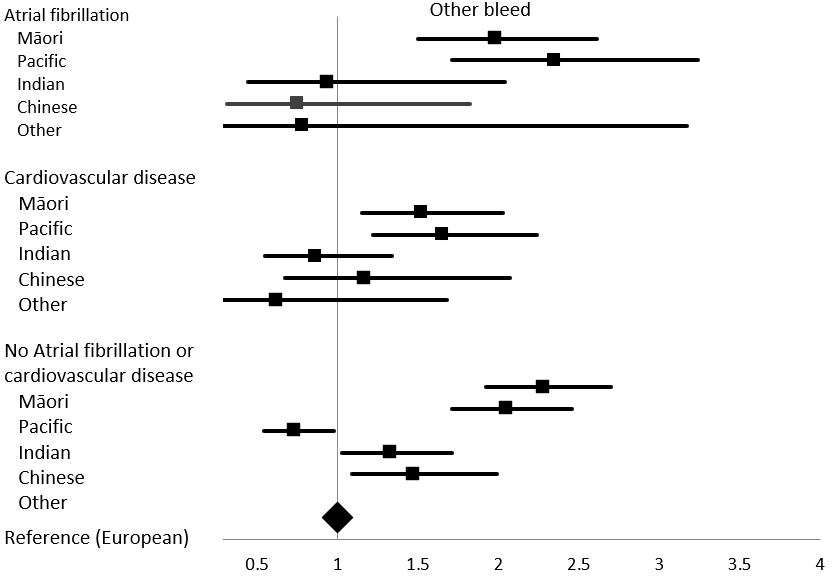


Hazard ratios are adjusted for all of the following variables: age, sex, ethnicity, socioeconomic deprivation, systolic blood pressure (>/= or < 140 mm Hg), ratio of total cholesterol to high density lipoprotein cholesterol (mmol/L), medical history (smoking, diabetes, cardiovascular disease, heart failure, cancer, bleeding, peptic ulcer disease, thrombocytopaenia, anaemia, chronic kidney disease, liver disease, pancreatitis or alcohol-related condition) and medication use in the preceding 6 months (antiplatelet, anticoagulant, BP-lowering, lipid-lowering, non-steroidal anti-inflammatory, steroid, selective serotonin re-uptake inhibitor).

The total number of people included (and excluded due to a missing value) in the models for atrial fibrillation, cardiovascular disease and no atrial fibrillation or cardiovascular disease were: 15097 (115), 43437 (353) and 425668 (3437), respectively.

For the atrial fibrillation subgroup, the number of bleeds in the models were: any (1202), gastrointestinal (723), intracranial (172), other (379). For the cardiovascular disease subgroup, the number of bleeds in the models were: any (2092), gastrointestinal (1470), intracranial (270), other (423). For the no atrial fibrillation or cardiovascular disease subgroup, the number of bleeds in the models were: any (6478), gastrointestinal (4482), intracranial (926), other (1196).
